# Multiplatform molecular profiling identifies potentially targetable biomarkers in malignant phyllodes tumors of the breast

**DOI:** 10.18632/oncotarget.6421

**Published:** 2015-11-28

**Authors:** Zoran Gatalica, Semir Vranic, Anatole Ghazalpour, Joanne Xiu, Idris Tolgay Ocal, John McGill, Ryan P. Bender, Erin Discianno, Aaron Schlum, Souzan Sanati, Juan Palazzo, Sandeep Reddy, Barbara Pockaj

**Affiliations:** ^1^ Caris Life Sciences, Phoenix, AZ, United States of America; ^2^ Department of Pathology, University Clinical Center Sarajevo, Sarajevo, Bosnia and Herzegovina; ^3^ Mayo Clinic, Scottsdale, AZ, United States of America; ^4^ Miraca Life Sciences, Phoenix, AZ, United States of America; ^5^ Division of Anatomic and Molecular Pathology, Washington University School of Medicine, Saint Louis, MO, United States of America; ^6^ Department of Pathology, Anatomy, and Cell Biology, Jefferson Medical College, Philadelphia, PA, United States of America

**Keywords:** breast, fibroepithelial tumors, malignant phyllodes tumor, biomarkers, molecular profiling

## Abstract

Malignant phyllodes tumor is a rare breast malignancy with sarcomatous overgrowth and with limited effective treatment options for recurrent and metastatic cases. Recent clinical trials indicated a potential for anti-angiogenic, anti-EGFR and immunotherapeutic approaches for patients with sarcomas, which led us to investigate these and other targetable pathways in malignant phyllodes tumor of the breast. Thirty-six malignant phyllodes tumors (including 8 metastatic tumors with two cases having matched primary and metastatic tumors) were profiled using gene sequencing, gene copy number analysis, whole genome expression, and protein expression. Whole genome expression analysis demonstrated consistent over-expression of genes involved in angiogenesis including *VEGFA, Angiopoietin-2, VCAM1, PDGFRA*, and *PTTG1*. EGFR protein overexpression was observed in 26/27 (96%) of cases with amplification of the *EGFR* gene in 8/24 (33%) cases. Two *EGFR* mutations were identified including EGFRvIII and a presumed pathogenic V774M mutation, respectively. The most common pathogenic mutations included *TP53* (50%) and *PIK3CA* (15%). Cases with matched primary and metastatic tumors harbored identical mutations in both sites (*PIK3CA/KRAS* and *RB1* gene mutations, respectively). Tumor expression of PD-L1 immunoregulatory protein was observed in 3/22 (14%) of cases. Overexpression of molecular biomarkers of increased angiogenesis, EGFR and immune checkpoints provides novel targeted therapy options in malignant phyllodes tumors of the breast.

## INTRODUCTION

Phyllodes tumors (PT) of the breast are rare, biphasic (fibro-epithelial) neoplasms, constituting ≤ 1% of all breast cancers [1], and are histologically classified as benign, borderline or malignant. The malignant variant is characterized by an overgrowth of the malignant stromal (sarcomatous) component and constitutes ∼20% of all phyllodes tumors. There is a significant potential (∼22%) for both local recurrence and distant metastasis [1]. These tumors may also pose a potential therapeutic challenge as no effective targeted therapy has been reported yet [1, 2].

Previous studies of the molecular genetics of PT showed the importance of the *TP53* gene and p53 protein in progression from benign to malignant phyllodes tumors [3–6]. A gene expression study conducted by Vidal et al. [7] revealed activation of gene clusters related to hypoxia and angiogenesis in malignant phyllodes cases. Expression of various angiogenic factors including CD34, CD105, vascular endothelial growth factor (VEGF) and hypoxia-inducible factor-alpha has also been reported in malignant phyllodes tumors [8–11]. Tse et al. [12] also found the tumor angiogenesis (measured by microvessel density) as an independent predictor of malignancy in phyllodes tumors. Recent gene sequencing studies revealed the presence of variety of mutations in malignant phyllodes tumors of which *MED12* [Mediator Complex Subunit 12] mutation appears to be the most consistent and shared by both malignant and benign fibroepithelial tumors [13–15]. However, truly actionable (targetable by specific drugs) genetic alterations in PT appear to be rare. Majority of the previous studies showed EGFR protein overexpression and *EGFR* gene amplification in PT [13, 16–18]; However, Tse et al. [19] reported a low *EGFR* amplification rate (8%) in EGFR protein positive phyllodes tumors. No targetable *EGFR* activating mutations have been reported thus far.

Recently, cancer immunoediting involving immune check point proteins programmed cell death-1 (PD-1/CD279) expressed on tumor infiltrating T-lymphocytes and its ligand (PD-L1/CD274) expressed on tumor cells have come into the clinical focus due the remarkable therapeutic benefits caused by their specific inhibitors in patients with advanced melanoma, renal cell carcinoma and non-small cell lung carcinoma [20–21]. PD-1/PD-L1 expression was also reported in soft tissue sarcomas, which indicated a potential for targeting this pathway in a variety of cancers [20, 22–26].

In the present study, we comprehensively profiled a series of malignant phyllodes tumors of the breast in an attempt to identify potentially targetable pathways/biomarkers.

## RESULTS

### Patients

The study included 36 malignant (high grade) phyllodes tumors of which 24 were primary, 8 metastatic and 4 recurrent malignant phyllodes tumors of the breast (Table 1). Two metastatic phyllodes cases (lung metastasis) had their primary site samples available for molecular testing. All patients were females with the mean age of 50.8 years (range, 17–76 years).

**Table 1:** Results of multiplatform molecular profiling of 36 malignant phyllodes cases

Case (site)	PD-L1 protein (IHC)	EGFR protein (IHC)	*EGFR* (ISH)	*EGFR* (mutations)	NGS and Sanger (other mutations)
**Case#1 (P)**	Negative	Positive	Negative	Wild type	TP53 (G245S) PIK3CA (H1047R) PIK3CA (VUS G106_R108del)
**Case#2 (P)**	n/a	Positive	Negative	Wild type	None
**Case#3 (P)**	Negative	Positive	Negative	Wild type	PIK3CA (H1047R) PIK3CA (E545K)
**Case#4 (P)**	Negative	Positive	Negative	Wild type	None
**Case#5 (P)**	Negative	Positive	Negative	Wild type	TP53 (R248Q)
**Case#6 (P)**	Negative	Positive	Negative	Wild type	RET (VUS S653T)
**Case#7 (P)**	Negative	Positive	Negative	EGFR (V774M)	None
**Case#8 (P)**	Negative	Positive	Negative	Wild type	PIK3CA (H1047L)
**Case#9 (P)**	Negative	Positive	Amplified	Wild type	n/a
**Case#10 (P)**	Positive	Positive	Negative	Wild type	BRCA1 (M17751) BRCA2 (A371T, VUS)
**Case#11 (P)**	Negative	Positive	Amplified	Wild type	n/a
**Case#12 (R)**	Negative	Positive	n/a	Wild type	n/a
**Case#13 (P)**	Negative	Positive	n/a	Wild type	n/a
**Case#14 (P)**	Positive	Negative	Negative	Wild type	n/a
**Case#15 (P)**	Positive	Positive	Amplified	Wild type	TP53 (R248W) CDH1 (Q422K VUS)
**Case#16** **P** **M**	Negative Positive	Positive Positive	Negative Negative	Wild type Wild type	PIK3CA (H1047L), KRAS (G12D) PIK3CA (H1047L), KRAS (G12D)
**Case#17** **P** **M**	Negative Positive	Positive Positive	Negative Negative	Wild type Wild type	RB1 (P347fs) RB1 (P347fs)
Case#18 (R)*	n/a	n/a	n/a	n/a	n/a
Case#19 (M)*	n/a	n/a	Amplified	Wild type	n/a
Case#20 (P)*	n/a	Positive	n/a	n/a	TP53 (T140fs) (Y234C)
Case#21 (P)*	n/a	n/a	negative	Wild type	None
Case#22 (M)*	n/a	n/a	Amplified	Wild type	TP53 (R282W)
Case#23 (R)*	n/a	Positive	negative	Wild type	TP53 (G245S)
Case#24 (P)*	n/a	Positive	Amplified	EGFRvIII	n/a
Case#25 (P)*	n/a	Positive	Amplified	Wild type	TP53 (D281V)
Case#26 (M)*	n/a	Positive	Negative	Wild type	TP53 (R175H)
**Case#27 (M)**	n/a	Positive	Negative	Wild type	TP53 (V157F)
**Case#28 (P)**	n/a	Positive	n/a	Wild type	None
**Case#29 (P)**	n/a	Positive	n/a	Wild type	None
**Case#30 (R)**	n/a	n/a	n/a	n/a	None
**Case#31 (P)**	n/a	Positive	n/a	Wild type	TP53 (S99fs)
**Case#32 (M)**	n/a	n/a	Negative	Wild type	TP53 (R175H)
**Case#33 (P)**	Positive	n/a	n/a	Wild type	MLH1
**Case#34 (P)**	Positive	Positive	n/a	Wild type	TP53 (L194R) ATM (T616I)
**Case#35 (P)**	Positive	Positive	Amplified	Wild type	TP53 (c.560–23_561del) TP53 (R273H)
**Case#36 (M)**	Negative	Positive	n/a	Wild type	n/a

* Gene expression profiling

IHC – immunohistochemistry; ISH – *in situ* hybridization; NGS – next-generation sequencing

P – Primary; R – recurrent; M – metastatic

n/a – not available

VUS – variant of unknown significance

### Tumor characteristics

#### EGFR status

**Figure 1:**
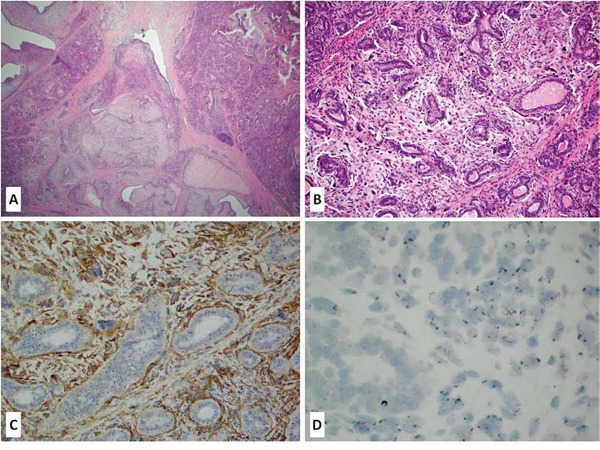
A-D: Primary malignant phyllodes tumor of the breast (A-B H&E stain, 10–20x magnification) with a strong membranous EGFR protein overexpression (C – IHC stain) accompanied by *EGFR* gene amplification (D – CISH)

EGFR protein overexpression (H-score ≥ 20) was observed in 26 of 27 tested cases (96%) while amplification of the *EGFR* gene was observed in 8 of 24 tested cases (33%); all *EGFR* amplified cases strongly overexpressed EGFR protein (H-score > 200) (Figure 1A–1D). *EGFRvIII* mutant variant was unequivocally detected in one out of 29 tested cases (∼3%); an additional case exhibited a borderline MLPA score (0.8). Both cases were primary phyllodes tumors overexpressing EGFR protein by IHC. Unequivocal *EGFRvIII* mutant case also harbored *EGFR* gene amplification. Activating *EGFR* gene mutation was observed in one primary phyllodes tumor (presumed pathogenic V774 mutation). This case also overexpressed EGFR protein without *EGFR* gene amplification or any other potentially targetable mutations.

#### Immune check-point proteins (PD-1 and PD-L1)

**Figure 2:**
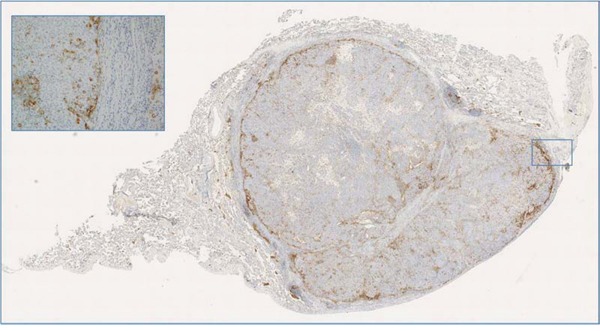
A case of metastatic phyllodes tumor to the lung with peripheral PD-L1 expression adjacent to the inflammatory cells and normal lung parenchyma; of note this case harbored *RB1* gene mutation in both primary and metastatic tumor

PD-1 and its ligand PD-L1 were evaluated in 22 phyllodes cases. Eight cases (36%) exhibited scattered, below threshold PD-L1 positivity on neoplastic cells while 3 cases including one metastatic phyllodes tumor showed positivity above the 5% threshold. In some cases PD-L1 expression was also detected on mono-nuclear cells within and surrounding the tumor (Figure 2). PD-1 positive TILs were completely absent in 12 cases, while in other cases varied in density from 1/10 hpf to > 100/10 hpf.

#### Mutational profile

NGS and/or Sanger sequencing assays were successful in 27 cases (Table 1). Apart from the above described *EGFR* mutations, several other genes were mutated including: *TP53* and, *PIK3CA* (affecting 50% and 15% of cases respectively), and *BRCA1, BRCA2, RET, CDH1, MLH1, ATM, KRAS,* and *RB1* (all affecting single phyllodes cases; Table 1). Of note, two cases with matching primary and metastatic (lung) samples harbored identical mutations in both sites: case#1 affecting a 56-year-old female had *PIK3CA* (H1047L) and *KRAS* (G12D) while case#2 (43-year-old female) harbored *RB1* (P347fs) gene mutation. Of note, no mutations in *KDR* (*VEGFR2*) were detected (*n* = 27).

#### Whole genome expression profiling

**Figure 3:**
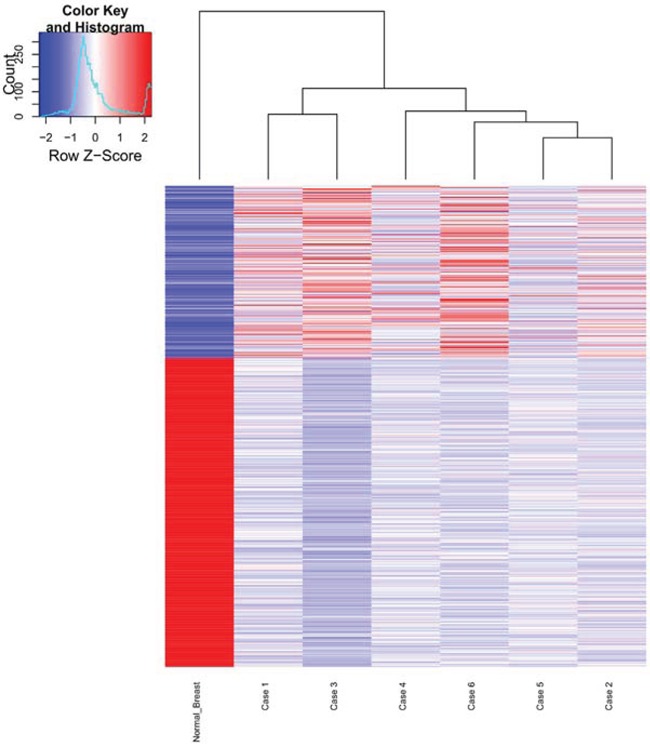
Gene expression signature of six phyllodes cases along with the normal breast tissue In the heatmap, rows represent genes and columns represent samples. “Upregulated” (depicted in red) is defined as a transcript with transcript level that is > 2 fold relative to normal breast control and down regulated (depicted in blue) is defined as a transcript with transcript level that is < 2 fold relative to control. The expression of the normal breast is shown in the far left column.

Differential expression analysis of the mRNA as measured by the Illumina array platform was carried out by comparing the normalized expression of the transcripts to the expression of the genes in the normal breast tissue. At the two fold cutoff, 247 probes representing 243 genes were found to be up regulated and 443 probes representing 422 genes were found to be down regulated (Figure 3, Supplementary Table 1). List of upregulated genes includes the 57 probes (57 genes) and 19 probes (18 genes) that were upregulated by 5 fold. The list of significantly enriched Gene Ontology (GO) terms included terms associated with GTPase activity, cytokine binding, and several cell division terms.

**Figure 4:**
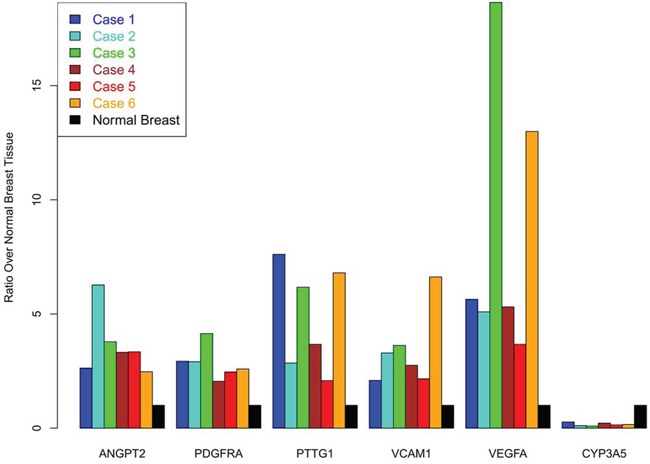
Bar plots for 6 angiogenesis markers found to be differentially regulated in Phyllodes cases when compared to normal breast tissue The height represents the ratio of expression for the gene in the phyllodes case over the expression in the normal breast. For CYP3A5, three phyllodes cases had no detectable expression of the transcript and the values are depicted as 0 ratio. Normal breast expression ratio is set to ‘1’ for all 6 biomarkers and is depicted as the ‘black’ bar for all 6 genes.

We found that 6 angiogenic markers (*VEGFA, Angiopoietin-2, VCAM1, PDGFRA, PTTG1*, and *CYP3A5*) were differentially expressed by at least 2 fold in phyllodes patients when compared with control (Figure 4, Supplementary Table 1). Moreover, our list significantly overlapped with the “angiome” list as published by Rivera et al. [31] (17 common genes, hypergeometric test *p*-value = 0.005).

## DISCUSSION

Malignant phyllodes tumor of the breast is a rare malignancy (<1%) with a substantial potential for both local relapse and distant metastasis (∼20%) [1]. Apart from surgery, no optimal treatment modalities with impact on overall survival are available at present [32]. Several recent studies indicated that molecular profiling of such “orphan tumors” may identify actionable targets with a successful outcome, even in metastatic setting [13, 22, 33–36].

The present study using comprehensive, multiplatform molecular approach revealed several potentially targetable pathways/biomarkers in these neoplasms. We found upregulation of several angiogenesis-related genes including *VEGFA, Angiopoietin-2, VCAM1, PDGFRA*, and *PTTG1* [16–19]. These findings may be clinically relevant as the upregulation of proangiogenic genes may predict response to the targeted therapy (e.g. angiogenesis inhibitors). We recently reported on a recurrent mammary angiosarcoma that was successfully treated with anti-angiogenic drug sunitinib, identified as a potentially beneficial therapy option using the whole genome array analysis [34]. In addition, anti-angiogenic targeted therapies (e.g. tyrosine kinase inhibitors, monoclonal antibodies) are promising therapeutics for the treatment of a wide spectrum of soft tissue tumors [37]. We also found overexpression of EGFR protein (with or without *EGFR* gene amplification or mutations), which could lead to the use of dual VEGFR/EGFR inhibitors (e.g. Vandatenib) or a combination of anti-VEGFR (e.g. bevacizumab) and anti-EGFR (e.g. erlotinib/gefitinib/cetuximab) therapies [38, 39]. Overexpression of PDGFRA would suggest the potential study of pazopanib, a multikinase inhibitor, with c-KIT, FGFR, PDGFR and VEGFR being amongst the inhibited enzymes. Of note, we report here for the first time that EGFRvIII (a mutant form of EGFR with deletion of exons 2–7 of the gene) may play a role in a small subset of malignant phyllodes tumors, adding this cancer to the growing list of malignancies with this type of mutation [40], and potentially eligible for tumor directed immunotherapy.

The selective mutational profiling (TSACP) of malignant phyllodes tumors revealed that *TP53* and *PIK3CA* gene mutations are common. These results are in line with previous data (COSMIC database, accessed on July 21, 2015; [41]). In contrast to the study of Tsang et al. [2] who also sequenced two matched phyllodes cases without recurrent mutations (*SMAD4, IDH1, PIK3CA, RET, TP53*), we found identical mutational profile in both primary and metastatic phyllodes cases (*PIK3CA/KRAS* and *RB1* gene mutations, respectively). *PIK3CA* and *RB1* alterations have been already described in phyllodes tumors [2, 13, 42] while Tsang et al. [2] found *HRAS* and Jardim et al. [33] *NRAS* in a borderline, and metastatic phyllodes tumors, respectively. The frequency of *PIK3CA* gene mutations in our study (15%) is the highest in comparison with the previous data [2] and this finding may imply a potential therapeutic benefit of the treatment with mTOR inhibitors.

Recently reported expression of the two immune check point targetable proteins PD-1 and PD-L1 in a variety of solid tumors including some sarcomas [20–26], led us to investigate them in this cohort of PT. Here we found that overexpression of PD-L1 characterizes a small subset of malignant phyllodes tumors including some metastatic cases, which may lead to the targeted immunotherapy for these patients. Numerous studies have reported remarkable benefits from PD-1/PD-L1 blockade in patients with other malignancies over-expressing PD-L1 (e.g. renal cell carcinoma, NSCLC, malignant melanoma, [21, 43–44]).

Limitations of our study are related to its retrospective design and the small number of cases tested by the Illumina microarray assay. Also, further prospective studies should confirm the clinical relevance of profiling-identified biomarkers.

In conclusion, this study provides additional support for comprehensive profiling in PT, which can identify several potentially targetable pathways including EGFR, angiogenesis, and immunotherapy for patients with locally advanced or metastatic tumors.

## MATERIALS AND METHODS

### Tissue samples

Thirty-six formalin-fixed paraffin-embedded (FFPE) samples of malignant phyllodes tumors were profiled at Caris Life Sciences, Phoenix, AZ (Molecular Intelligence Service^™^). All samples were previously diagnosed by referring pathologists and histologic diagnosis was confirmed centrally by a board certified pathologist.

### Immunohistochemistry (IHC)

Expression of EGFR, PD-1 and PD-L1 was evaluated immunohistochemically using commercially available antibodies and detection kits [EGFR (Invitrogen); PD-1, (NAT1 antibody, Cell Marque); anti-PD-L1 (SP142, Spring Bioscience)]. The extent of EGFR expression was evaluated using H-score (intensity of staining graded on a subjective scale of 0–3 and percentage of cells with given intensity were multiplied and added up to provide an H-score, which ranged from 0–300). Tumor infiltrating lymphocytes (TIL), which expressed PD-1 on their plasma membrane, were evaluated and their density (number of PD-1+ TILs per high power field) was recorded. Membranous expression of PD-L1 in more than 5% of tumor cells was considered positive [20, 22–23, 27]. All IHC assays included positive and negative controls to support the validity of results.

### *EGFR* gene alterations

#### Copy number changes: *In-situ* hybridization (fluorescent and chromogenic ISH)

FISH [*EGFR/CEP7* probe] (Abbott Molecular/Vysis) and CISH (dual EGFR DNP/CEP 7 DIG probes, Ventana, Tucson, AZ) assays were used for evaluation of the *EGFR* gene status. *EGFR* gene was considered amplified if EGFR/CEP7 ratio > 2, or > 15 *EGFR* gene copies per cell were observed in > 10% of analyzed cells [28].

### *EGFRvIII* mutation: fragment analysis (FA) sequencing and multiplex ligation-dependent probe amplification (MLPA)

Mutation analysis for EGFRvIII was performed on RNA extracted from FFPE tissue in 18 cases. Two sets of FAM linked primers were used to PCR amplify both the wild type and mutant EGFR alleles and PCR products were visualized using an ABI 3500xl. Signal generated from the wild type allele was used as an amplification control and samples were considered positive if EGFRvIII was detected at a level that is 5x typical background observed. Samples with EGFRvIII signals between 1–5 x standard background were considered indeterminate and < 1x standard background was considered a negative result. This assay requires samples to have at least 50% tumor nuclei [29].

Eleven PTs were evaluated for EGFRvIII status using a multiplex ligation-dependent probe amplification (MLPA) assay (SALSA MLPA KIT P315-A1 EGFR, MRC-Holland kit, MRC-Holland, Amsterdam, Netherlands). Values between 0.8 - 1.5 were considered normal, while values < 0.8 as a loss, > 1.5 as a gain and values > 2 as an amplification.

### Sequencing analysis (NGS and sanger sequencing)

NGS was performed on genomic DNA isolated from FFPE samples using the Illumina MiSeq platform. Specific regions of the genome were amplified using the Illumina TruSeq Amplicon - Cancer Panel (TSACP). The NGS panel included 46 genes (available here: http://www.carismolecularintelligence.com/next-generation-sequencing-profile). All variants were detected with > 99% confidence based on the frequency of the mutation present and the amplicon coverage using a mutation frequency threshold of 10% [23, 30]. All regions that were sequenced achieved a minimum of 100x coverage and overall samples had an average coverage of > 500x; most samples achieving 1000–2000x average coverage. Sanger Sequencing for selected regions of *BRAF, KRAS, c-KIT, EGFR*, and *PIK3CA* was also used.

### Microarray assays

For seven cases the whole-genome expression was analyzed using Illumina cDNA-mediated annealing, selection, extension and ligation (DASL) process with the HumanHT-12 v4 beadChip (Illumina Inc., San Diego, CA). Of the seven cases run on this platform one case was excluded from analysis due to overall low scanning intensity. The 6 remaining phyllodes cases were normalized using mean expression normalization where each array data was adjusted to have the same mean as the control breast microarray. The ratios of expression for each gene were calculated by dividing the normalized expression of the gene to the control breast tissue. The detection *p*-value of 0.001 was used to assess if a gene is expressed or not. The detection *p*-value is an output of the Genome Studio software (Illumina) and represents the confidence that a given transcript is expressed above the background defined by negative control probes on the array. All the data analysis for gene expression was carried out using the Genome Studio software and the R Software downloaded from CRAN website (http://cran.r-project.org).

## SUPPLEMENTARY TABLE




