# Supplementary material for: Multiplatform molecular profiling identifies potentially targetable biomarkers in malignant phyllodes tumors of the breast
Source: Oncotarget. 2015 Nov 28;7(2):1707–16. doi: 10.18632/oncotarget.6421 (PMC4811491; doi:10.18632/oncotarget.6421)
Supplement: Supplementary file 1 [file oncotarget-07-1707-s001.pdf]

SUPPLEMENTARY TABLE

Supplementary Table S1: List of significantly (2-fold) up- and downregulated genes in malignant phyllodes tumors of the breast.
